# Correction: Automated robotic arm system for real-time multi-parameter quality assessment of raw milk in cheese manufacturing

**DOI:** 10.1371/journal.pone.0355949

**Published:** 2026-08-11

**Authors:** Nadun Salinda, Tharaga Sharmilan

The following information is missing from the Acknowledgement statement: The authors would like to thank the LK Domain Registry for its publication fee support.
