# Supplementary material for: Automated robotic arm system for real-time multi-parameter quality assessment of raw milk in cheese manufacturing
Source: PLoS One. 2026 May 18;21(5):e0346027. doi: 10.1371/journal.pone.0346027 (PMC13183210; doi:10.1371/journal.pone.0346027)
Supplement: Attachment — Submitted filename: Response table.docx [file pone.0346027.s002.docx]

Dear Editor and Reviewers,
We thank you for your valuable feedback. All comments have been carefully addressed, and corresponding revisions have been incorporated into the manuscript.

A detailed summary of the actions taken is provided in the table below for clarity.

Sincerely
Dr Tharaga Sharmilan
Corresponding Author

| **Reviewer 01** | |
| --- | --- |
| 1.1 Background | |
| The authors rightly emphasize the importance of milk quality, but the literature review is relatively narrow. There is a lack of references to studies from Europe or North America, which also investigate automation systems in the dairy sector. | 3 citations have been added |
| 1.2 Automation in dairy quality assessment | |
| Citations ([16], [13]) are used correctly, but there is no critical evaluation of the limitations of these systems—the authors mostly list them briefly. The narrative about “IoT-enabled systems” is repeated without presenting actual numerical data (e.g., the accuracy achieved by other systems). | Section 1.2-line numbers 70-86 |
| 1.4 Gaps in current research | |
| The identification of research gaps is convincing, but the authors fail to address issues related to implementation costs and the long-term reliability of sensors. | Updated -Section 1.4-line numbers 129-131 |
| 2.1 Research design | |
| There is a contradiction: at the beginning the paper states that milk was classified using threshold logic, but only later (section 2.8) introduces SVM. The narrative needs clarification-is the study comparing both approaches, or was threshold logic only a baseline? | Clearly mentioned, section 2.1 line numbers 163-165. |
| 2.3 Sensors and measurement principles | |
| The reported pH threshold range (6.6–8.8 for “fresh milk”) seems too wide—typical fresh milk has a pH of ~6.6–6.8. Including 8.8 as the upper boundary raises methodological concerns. This subsection also lacks details on sensor calibration procedures, which are critical for field studies. | Table 1-pH upper limit is 6.8. 8.8 is a mistake. It is now updated.   section 2.3 -line number 219-221 |
| 2.4 Mechanical design | |
| There is an inconsistency: the introduction mentions a “six-degree-of-freedom robotic arm,” whereas this section describes a “four-DOF robotic arm.” This is a significant discrepancy that needs clarification | It is now updated. Four DoF |
| 2.6 Testing protocol 2.6.1 Laboratory repeatability | |
| Only 30 powdered milk samples were tested, which does not reflect the real variability of fresh milk. Please provide test powder | Updated now-Section 2.6.1 |
| 2.6.2 Fresh milk performance tests |  |
| The sample size (n=30; 15 cow, 15 buffalo) is too small to draw strong statistical conclusions. Please provide test power. | It is now updated. section 2.6.2 |
| 2.6.3 Field validation |  |
| Tests were conducted at only three collection centers with five samples per site-a very limited dataset | Section 2.6.3- 396-399 |
| 2.8 Machine learning-based classification |  |
| Training on only 90 samples is insufficient for SVM; there is a clear risk of overfitting. The authors mention 10-fold CV but report only “accuracy” and “F1-score.” Other metrics such as AUC, confusion matrix from cross-validation, or comparisons with alternative models (RF, NN) are missing. | Section 2.8-line numbers 452-560  Section 3.5-line numbers 641-645 |
| Results 3.1 Sensor accuracy |  |
| Temperature readings showed large deviations (-8.8°C from the reference value)—a serious practical issue, yet the authors discuss it very briefly. This section does not explore potential solutions nor report recalibration attempts. | Section 3.1-line numbers 536-539 |
| 3.2 RGB repeatability |  |
| The results are acceptable, but the test was conducted under controlled conditions only. There is no analysis of variability in field conditions. If such tests were not feasible, the authors should indicate this as a direction for future work. | Section 3.2 Line numbers 570-572 |
| 3.3-3.5 Field trials & Classification |  |
| The results are promising (accuracy ~97%), but the number of field samples (n=45) is insufficient. Please provide test power. The class distribution (number of fresh vs. spoiled samples) is not reported. Sensitivity and specificity are missing—accuracy alone may be misleading if the dataset is imbalanced. | Section 3.3-line numbers 600-604 |
| **Reviewer 02** | |
| The manuscript does not contain page numbers or line numbers, making it very difficult to provide precise comments. Please add both for ease of review. | Line numbers and page numbers are included now. |
| There are many repeated sentences throughout the manuscript, particularly regarding measurement accuracy. These repetitions should be removed or consolidated. | It is updated in Abstract line number 19-20, Discussion line numbers 624-625. |
| The manuscript claims significant time savings, but parameters such as pH and temperature can already be measured very quickly using available instruments. Therefore, the claim of time savings for these two parameters is not justified and should be reconsidered or supported with stronger evidence | Abstract line numbers 20-24 section 3.4 line numbers 562-566 section 4 line numbers 620-624 section 4 line numbers 661-662. |
| . Color Measurement Concern: The color sensor used in the study does not appear capable of providing accurate color characterization. Thus, determining milk quality solely based on this color data is questionable | Section 2.3 line numbers 234-237. section 3.2 line numbers 516-518  Section 4 line numbers 639-641. |
| The power source required for operating the device is not clearly explained. This could be a major concern for deployment in different environments. Please clarify the power requirements and portability | Section 2.10 line numbers 461-471 |
| English language requires improvement. Several sentences need clearer explanation. | Thoroughly checked and refined |
| The reference numbering is inconsistent. The first citation in the manuscript should appear as [1], with subsequent references following sequentially. For example, in Section 1.2, the second paragraph cites [16] and [13] prematurely. Please revise the entire referencing system. | It is now updated |
| Table 1: The labels “density of fresh cow milk fresh” and “buffalo milk fresh” should be corrected to: “Cow Milk (Fresh)”; “Buffalo Milk (Fresh)” | Bracket is used -Table 1 |
| Table 4: The measured temperature values should be added, along with the corresponding mean accuracy. | Table 4 -3^rd^ column |

**Editors’ Comments**

| 1. Please ensure that your manuscript meets PLOS ONE's style requirements, including those for file naming. The PLOS ONE style templates can be found at <https://journals.plos.org/plosone/s/file?id=wjVg/PLOSOne_formatting_sample_main_body.pdf> and <https://journals.plos.org/plosone/s/file?id=ba62/PLOSOne_formatting_sample_title_authors_affiliations.pdf> | Re checked them |
| --- | --- |
| In your Methods section, please provide additional information regarding the permits you obtained for the work. Please ensure you have included the full name of the authority that approved the field site access and, if no permits were required, a brief statement explaining why | Section 2.6.3-line numbers 408-412 |
| When completing the data availability statement of the submission form, you indicated that you will make your data available on acceptance. We strongly recommend all authors decide on a data sharing plan before acceptance, as the process can be lengthy and hold up publication timelines. Please note that, though access restrictions are acceptable now, your entire data will need to be made freely accessible if your manuscript is accepted for publication. This policy applies to all data except where public deposition would breach compliance with the protocol approved by your research ethics board. If you are unable to adhere to our open data policy, please kindly revise your statement to explain your reasoning and we will seek the editor's input on an exemption. Please be assured that, once you have provided your new statement, the assessment of your exemption will not hold up the peer review process. | We accepted. |
| 4. We note that Figure(s) 2, 3, 4, 5, in your submission contain copyrighted images. All PLOS content is published under the Creative Commons Attribution License (CC BY 4.0), which means that the manuscript, images, and Supporting Information files will be freely available online, and any third party is permitted to access, download, copy, distribute, and use these materials in any way, even commercially, with proper attribution. For more information, see our copyright guidelines: <http://journals.plos.org/plosone/s/licenses-and-copyright>. We require you to either (1) present written permission from the copyright holder to publish these figures specifically under the CC BY 4.0 license, or (2) remove the figures from your submission: You may seek permission from the original copyright holder of Figure(s) 2, 3, 4, 5, to publish the content specifically under the CC BY 4.0 license. | This is our own research specifically figures.  Therefore, We are permitting to publish these figures specifically under the CC BY 4.0 license. |
| We recommend that you contact the original copyright holder with the Content Permission Form (<http://journals.plos.org/plosone/s/file?id=7c09/content-permission-form.pdf>) and the following text: “I request permission for the open-access journal PLOS ONE to publish XXX under the Creative Commons Attribution License (CCAL) CC BY 4.0 (<http://creativecommons.org/licenses/by/4.0/>). Please be aware that this license allows unrestricted use and distribution, even commercially, by third parties. Please reply and provide explicit written permission to publish XXX under a CC BY license and complete the attached form.” Please upload the completed Content Permission Form or other proof of granted permissions as an "Other" file with your submission. | Permission letter is attached |
| In the figure caption of the copyrighted figure, please include the following text: “Reprinted from [ref] under a CC BY license, with permission from [name of publisher], original copyright [original copyright year].” | N/A |
| . If you are unable to obtain permission from the original copyright holder to publish these figures under the CC BY 4.0 license or if the copyright holder’s requirements are incompatible with the CC BY 4.0 license, please either i) remove the figure or ii) supply a replacement figure that complies with the CC BY 4.0 license. Please check copyright information on all replacement figures and update the figure caption with source information. If applicable, please specify in the figure caption text when a figure is similar but not identical to the original image and is therefore for illustrative purposes only. | N/A |
| If the reviewer comments include a recommendation to cite specific previously published works, please review and evaluate these publications to determine whether they are relevant and should be cited. There is no requirement to cite these works unless the editor has indicated otherwise. | All are updated |
| Please review your reference list to ensure that it is complete and correct. If you have cited papers that have been retracted, please include the rationale for doing so in the manuscript text, or remove these references and replace them with relevant current references. Any changes to the reference list should be mentioned in the rebuttal letter that accompanies your revised manuscript. If you need to cite a retracted article, indicate the article’s retracted status in the References list and also include a citation and full reference for the retraction notice. | Updated them |
